# Predictive value of C-reactive protein in critically ill patients after abdominal surgery

**DOI:** 10.6061/clinics/2017(01)05

**Published:** 2017-01

**Authors:** Frédéric Sapin, Patrick Biston, Michael Piagnerelli

**Affiliations:** Université Libre de Bruxelles, Intensive Care, CHU-Charleroi, 6042-Charleroi, Belgium

**Keywords:** C-reactive Protein, Biomarker, Abdominal Surgery, Outcome, Mortality, Antibiotics

## Abstract

**OBJECTIVES:**

The development of sepsis after abdominal surgery is associated with high morbidity and mortality. Due to inflammation, it may be difficult to diagnose infection when it occurs, but measurement of C-reactive protein could facilitate this diagnosis. In the present study, we evaluated the predictive value and time course of C-reactive protein in relation to outcome in patients admitted to the intensive care unit (ICU) after abdominal surgery.

**METHODS:**

We included patients admitted to the ICU after abdominal surgery over a period of two years. The patients were divided into two groups according to their outcome: favorable (F; left the ICU alive, without modification of the antibiotic regimen) and unfavorable (D; death in the ICU, surgical revision with or without modification of the antibiotic regimen or just modification of the regimen). We then compared the highest C-reactive protein level on the first day of admission between the two groups.

**RESULTS:**

A total of 308 patients were included: 86 patients had an unfavorable outcome (group D) and 222 had a favorable outcome (group F). The groups were similar in terms of leukocytosis, neutrophilia, and platelet count. C-reactive protein was significantly higher at admission in group D and was the best predictor of an unfavorable outcome, with a sensitivity of 74% and a specificity of 72% for a threshold of 41 mg/L. No changes in C-reactive protein, as assessed based on the delta C-reactive protein, especially at days 4 and 5, were associated with a poor prognosis.

**CONCLUSIONS:**

A C-reactive protein cut-off of 41 mg/L during the first day of ICU admission after abdominal surgery was a predictor of an adverse outcome. However, no changes in the C-reactive protein concentration, especially by day 4 or 5, could identify patients at risk of death.

## INTRODUCTION

Sepsis, defined as a clinically suspected or proven infection associated with an inadequate systemic immune response, is characterized by hemodynamic, metabolic, and both pro- and anti-inflammatory derangements. In less severe cases, these derangements are defined as non-specific signs (also called systemic inflammatory response syndrome, or SIRS). More severe cases, with dysfunction of at least one organ, are called severe sepsis or are termed septic shock if there is associated hypotension requiring vasopressor therapy to maintain the mean blood pressure at 65 mmHg or greater and if the serum lactate level is greater than 2 mmol/L 1,2. Although the intensive care unit (ICU) mortality rates for patients with severe sepsis or septic shock have decreased from 50% to 25-30% in recent years 3, these rates remain high, and there is also considerable morbidity related to the associated organ dysfunction 4. The decreased mortality over the years has perhaps been due to better awareness and recognition of the syndrome, resulting in more rapid institution of aggressive management, including fluid resuscitation, monitoring and appropriate empirical antibiotic therapy 3,5,6 as well as both identification of infectious foci and surgical intervention, if required.

Considerable effort is being placed on identifying biomarkers that can be used to detect sepsis in an early and reversible phase to decrease mortality from this disease 7. In the management of septic patients, it is also imperative to closely monitor the progression of the disease and to evaluate the likely prognosis because this information may influence individual clinical interventions 1. Therefore, there is a need for biomarkers that can help in the monitoring and staging of sepsis and, consequently, in guiding therapeutic management 7-9. Ideally, biomarker measurement should be easy, fast and inexpensive, and the results should have high specificity and sensitivity for sepsis. Several biomarkers have been proposed or are under investigation in the context of sepsis 7,10-13. In particular, C-reactive protein (CRP) measurements are already widely used for this purpose in critically ill patients 14.

Despite its routine clinical use, few studies have examined the CRP concentration as a biomarker of infection in critically ill patients, and the results have not been consistent. In a recent review of studies performed in critically ill patients, Lelubre et al. 14 suggested that the time course of CRP is more useful than a single measurement. However, the numbers of patients included in the studies were limited, the causes of sepsis were very heterogeneous, and few surgical ICU patients were included 14. In a meta-analysis, Zhang and Ni confirmed this study heterogeneity (I^2^=92%), limiting understanding of the contribution of CRP to prognosis 15.

The development of sepsis in critically ill patients after abdominal surgery, even if scheduled surgery, is associated with high morbidity and mortality 16. Due to persistent inflammation, it may be difficult to diagnose infection when it occurs 17. Despite its potential role as a biomarker of infection, few studies have assessed the usefulness of CRP in the diagnosis of sepsis after abdominal surgery in critically ill patients 18,19. For these reasons, we investigated the predictive value of the CRP concentrations on the first day of ICU admission compared to other inflammatory parameters and the time course of CRP in relation to ICU outcome, defined as favorable (discharged alive from the ICU, without modification of antibiotherapy) or unfavorable (modification of antibiotherapy during the ICU stay, a new abdominal surgical intervention for uncontrolled sepsis with or without modification of the initial antibiotic regimen, or ICU death), in critically ill patients admitted to the ICU after abdominal surgery.

## METHODS

This study was conducted in the 36-bed medicosurgical ICU of CHU-Charleroi, Belgium. We retrieved data for all adult (≥18 years) patients admitted to the ICU after major abdominal surgery between January 1, 2011, and December 31, 2012. Patients without complete biological or clinical data were excluded. The local ethical committee approved the study but waived the need for informed consent because of the retrospective nature of the study.

Demographic data at admission were recorded, including age, sex, height, weight, type of abdominal surgery, timing of surgery (scheduled or emergency), APACHE II 20 and SOFA 21 scores, length of ICU stay and ICU mortality. On the first day of the ICU stay, the following treatment and clinical data were collected: maximum dose of vasopressors, highest temperature, lowest mean blood pressure, lowest PaO_2_/FiO_2_, and diuresis. For each day during the ICU stay, we retrieved the platelet, white blood cell and neutrophil counts and the hemoglobin, fibrinogen, urea, creatinine, bilirubin, lactate and serum CRP concentrations. We calculated the delta CRP concentration as the highest value of CRP on a particular day minus the CRP concentration on the day of admission. Any antibiotherapy administered during the ICU stay was noted.

Hemodynamic management of these septic patients was in agreement with the Surviving Sepsis Campaign recommendations 1. All patients received prophylactic or empirical antibiotic therapy before surgery, in agreement with local policy. No protocol concerning ICU admission after major abdominal surgery was in place at our institution; nevertheless, we arbitrarily defined a patient with major abdominal surgery as a patient with hemodynamic instability during the surgery; with a transfusion requirement; or with significant comorbidities, such as impairment of the left ventricular ejection fraction, chronic obstructive pulmonary disease, or chronic renal failure. All signs of tissue hypoperfusion before or during surgery motivated admission to the ICU.

We defined a patient with a favorable ICU outcome as a patient who was discharged alive from the ICU, without modification of antibiotherapy. In contrast, an unfavorable outcome was defined as requiring a modification of antibiotherapy during the ICU stay, a new abdominal surgical intervention for uncontrolled sepsis with or without modification of the initial antibiotic regimen, or ICU death.

### Statistical analysis

Statistical analyses were performed using XLSTAT2013 (ADDINSOFT^®^). The data are presented as the median +/- IQR or the count (percentage), unless stated otherwise. All tests were two sided, and a *p*-value<0.05 was considered statistically significant. Comparisons between groups were performed using a Mann-Whitney test for continuous variables or Fisher’s exact test for dichotomous variables.

ROC curves were computed to compare the ability of inflammatory markers to predict an unfavorable outcome. Evolution of the CRP concentration over time and between groups was analyzed by ANOVA for repeated measures. Additionally, pairwise comparisons were performed using a Tukey test.

## RESULTS

**Figure 1:**
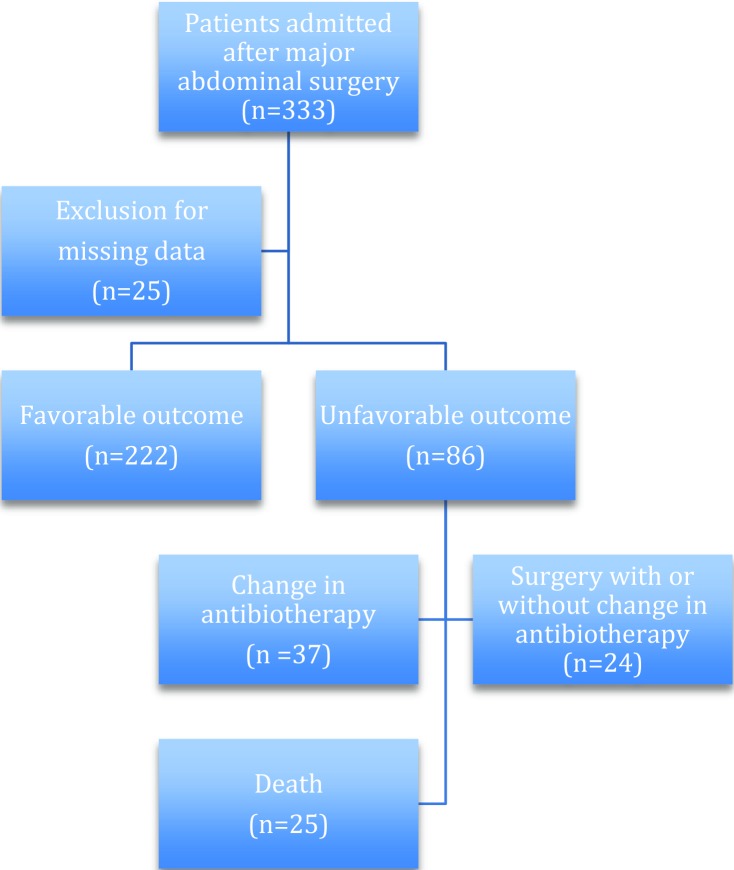
Flow chart of the patient selection.

During the 2-year study period, 4482 patients were admitted to our ICU. Of these, 333 patients (13.5%) were admitted after major abdominal surgery. After exclusion of 25 patients because of a lack of data (particularly no measurements of CRP concentrations), the final analysis included 308 patients (Figure 1).

The patient’s characteristics are summarized in Table 1. Their median age was 63 [52-72] years, and 50.6% were male. The median APACHE II score was 11 [6-16], and the median SOFA score was 3 [1-4]. In total, 14% of patients were treated with vasopressors on the first ICU day. The majority of the abdominal surgeries were colorectal (47%), and 132 (43%) surgeries were not planned. The median ICU length of stay was 3 [2-5] days, and ICU mortality was 8%. CRP concentrations were low on the first day of ICU admission for all patients included, with a median of 28 [7-163] mg/L.

We compared, on ICU admission, patients admitted for scheduled or emergency surgery. The patient’s characteristics are summarized in Table 2. As expected, ICU severity scores, ICU and hospital lengths of stay and mortality were higher among those with emergency surgery. Among the inflammatory markers examined, CRP concentrations were also higher in these patients, whereas the white blood cell count was not higher and neutrophilia was not more frequent (Table 2). A weak but significant correlation was observed between SOFA scores and CRP concentrations at ICU admission for all patients (r=0.27; *p*<0.0001).

Of the 308 patients, 222 (72%) had a favorable outcome (Figure 1). Among the remaining 86 patients with an unfavorable outcome, 25 (8% of the total population, 29% of the patients with an unfavorable outcome) died during their ICU stay. Mortality was significantly higher in those with emergency surgery than in those with scheduled surgery (18 *vs* 7 deaths, *p*=0.001). Empirical antibiotherapy was altered during the ICU stay for 37 patients (12% of the total population, 33% of the patients with an unfavorable outcome), and 24 patients (8% of the total population, 28% of the patients with an unfavorable outcome) needed a new abdominal surgical intervention with or without modification of their antibiotic regimen during their ICU stay (Figure 1).

Comparisons of clinical characteristics and biological data between patients with favorable and unfavorable outcomes are shown in Table 3. As expected, ICU severity scores and the ICU length of stay were higher in patients with an unfavorable outcome. In total, 28 patients in this subgroup (32.6%) were in septic shock (median dose of norepinephrine: 0.39 [0.17-0.58] *vs* 0.20 [0.12-0.29] mcg/kg/h for patients with a favorable outcome, *p*=0.024) and were more likely to have pulmonary (lower P/F: 220 [143-314] *vs* 315 [234-426], *p*<0.001) or renal (higher creatinine concentrations: 1.06 [0.80-2.00] *vs* 0.90 [0.70-1.12] mg/dL, *p*=0.002; higher urea concentrations: 51 [31-82] *vs* 32 [23-45] mg/dL, *p*<0.001) organ dysfunction and lower urinary output (850 [558-1290] *vs* 1100 [710-1670] mL, *p*=0.002) on the first ICU day. Lactate concentrations were also more elevated in the patients with an unfavorable outcome (Table 3).

Among the inflammatory biomarkers examined, only CRP values were significantly different between the groups: 13.6 [5-86] mg/L for those with a favorable outcome *vs* 140.5 [45.0-314.6] mg/L for those with an unfavorable outcome, *p*<0.001; Table 2).

We observed a weak correlation between ICU SOFA scores and CRP concentrations only in the group with an unfavorable outcome (r=0.28; *p*=0.01).

**Figure 2:**
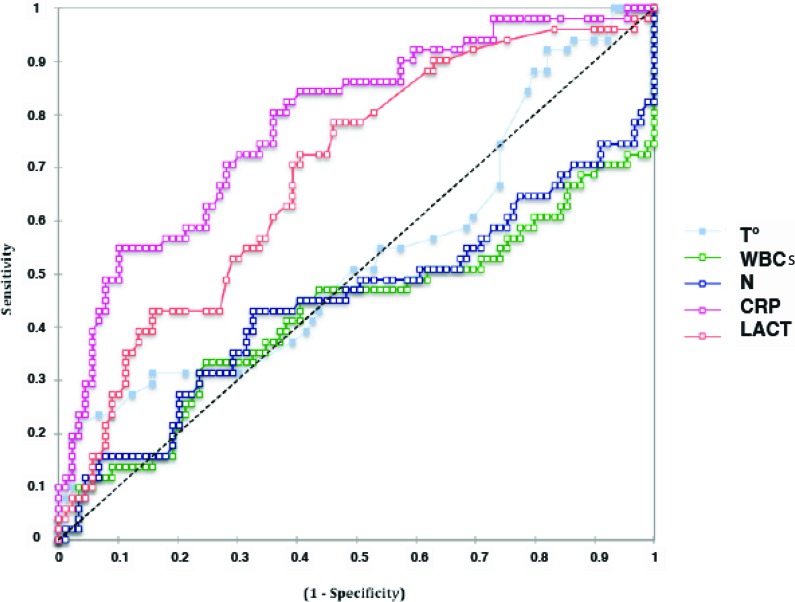
ROC curves for the measured inflammatory parameters at ICU admission for all patients. The area under the curve (AUC) values were as follows: CRP: 0.78 [0.71-0.84], *p***<**0.0001; lactate concentration (LACT): 0.73 [0.66-0.80], *p*<0.0001; leukocytosis (WBCs): (0.43 [0.32-0.53], *p*=0.18); neutrophilia (N): 0.43 [0.3-0.53], *p*=0.34; and temperature (T°): 0.53 [0.44-0.64], *p*=0.49.

CRP was the inflammatory marker that best predicted a poor outcome, with an area under the ROC curve of 0.78 [0.71-0.84] (*p*<0.0001; Figure 2). A CRP concentration threshold of 41 mg/L permitted prediction of ICU outcome with a sensitivity of 74% (62-83), a specificity of 72% (65-79), a positive likelihood ratio of 2.7, and a negative likelihood ratio of 0.37. After the CRP concentration, the lactate concentration had the highest area under the ROC curve (0.73 [0.66-0.80], *p*<0.0001; with a sensitivity of 82% and a specificity of 55% for a threshold of 15.6 mg/dL). In contrast, leukocytosis (AUC: 0.43 [0.32-0.53], *p*=0.18), neutrophilia (AUC: 0.43 [0.3-0.53], *p*=0.34) and temperature (AUC: 0.53 [0.44-0.64], *p*=0.49) did not have significant predictive value (Figure 2).

**Figure 3:**
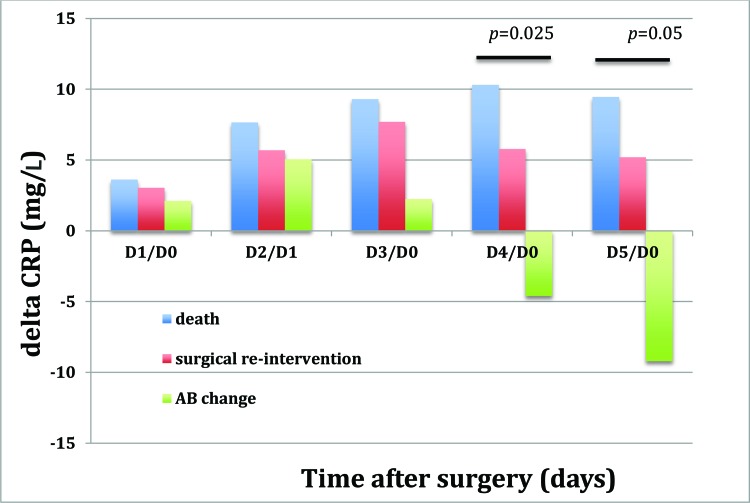
Changes in delta CRP in the patients with an unfavorable outcome (death, surgical re-intervention, or a change in antibiotic regimen; n=86). The evolution of the CRP concentration over time and between groups was analyzed using ANOVA for repeated measures.

For the 86 patients with an unfavorable outcome, we analyzed the delta CRP concentrations in the three subgroups (modification of the empirical antibiotic regimen, new surgery with or without modification of the antibiotic regimen and death) (Figure 3). There was a significant difference in the delta CRP for day 4/day 0 (*p*=0.025) and day 5/day 0 (*p*=0.05) between patients who only had modification of their antibiotic regiment and patients who died (Figure 3).

## DISCUSSION

Sepsis complicating major abdominal surgery is associated with high morbidity and mortality 16,30. Use of a biomarker, such as CRP, in addition to clinical signs may help clinicians to diagnose sepsis early and to start timely treatment.

In this study, we showed that CRP concentrations during the first ICU day were a good marker of outcome. In particular, a value greater than 41 mg/L had high specificity for an unfavorable outcome. Moreover, the time course of the CRP concentration, as assessed based on the delta CRP, differentiated between patients who died and those who only needed a change in their antibiotic treatment.

These findings have already been reported for ICU patients, and notably patients with community- or ventilator-acquired pneumonia 23,24, bacteremia 25,26 or hepatic failure 27,28. In contrast, in the present study, the CRP values were analyzed and related to outcome in a large cohort of ICU patients after abdominal surgery. Indeed, CRP has often been studied as a potential marker for diagnosis and/or the need to reoperate 19,29-32. For diagnosis of infection in ICU patients, however, the results of the various studies have been contradictory 14,18,19, but an increase in CRP levels has been described as a crucial indicator for the diagnosis of postoperative complications, such as infection, sepsis, anastomotic leakage or mesenteric ischemia 19,29-31. For example, CRP levels >140 mg/L on the 4th postoperative day after rectal surgery with primary anastomosis had a 91% predictive value for a complicated postoperative course 30. In contrast, in a recent systematic review and meta-analysis, Singh et al. reported that seven studies with a total of 2483 patients indicated the “usefulness” of CRP values as a negative predictive marker for the development of anastomotic leakage following colorectal surgery 32.

Regarding ICU patients after abdominal surgery, the number of studies is limited. Indeed, Schmit and Vincent 18 reported the time course of CRP concentrations in 50 septic patients with adequate (n=24) or inadequate (n=18) empiric antibiotherapy and in surgical patients who needed reoperation for uncontrolled infection (n=8). As expected, CRP concentrations decreased faster during the first 48 hours when the antibiotherapy was adequate, and an increase in the CRP concentration by a minimum of 22 mg/L over the 48-hour period was predictive of inadequate antibiotherapy, with a sensitivity of 77% and a specificity of 67% 18. The results were identical if a longer delay was allowed between CRP measurements. The take-home message of that study is the need for at least two CRP measurements separated by a delay of 48 hours to estimate the appropriateness of antibiotherapy, as also suggested by the meta-analysis by Zhang and Ni 15. Another interesting aspect of the aforementioned study 18 was the time course of CRP in a surgical population with uncontrolled infection. Regrettably, however, the number of patients studied was limited (n=8), and the delay to reoperation in cases with uncontrolled infection was not reported, limiting the conclusions that can be drawn regarding the usefulness of CRP values in this particular population 30.

In a prospective, monocentric observational study that included 174 surgical patients, Meyer et al. 19 reported that a 10% increase in the CRP concentration resulted in a 3.5% increase in the odds of an event (a diagnostic or therapeutic intervention; OR 1.035, 95% CI: 1.004-1.068, *p*=0.028). Nevertheless, an increase in the CRP level did not lead to higher odds of complications (OR 0.983, 95% CI: 0.932-1.036, *p*=0.52), even after adjustment for the SOFA score (OR 0.980, 95% CI: 0.929-1.035, *p*=0.46). Hence, the authors concluded that an increase in the CRP concentration was a poor marker of complications in surgical patients. There are several differences between our study and the study by Meyer et al. 19. For example, we included only patients admitted post-operatively after major abdominal surgery, whereas Meyer et al. included all surgical patients (especially vascular surgery patients) except those who had cardiac surgery. Moreover, more than 10% of their patients were admitted to the ICU for sepsis 19, and a large number of these patients were admitted following re-exploration for complications from earlier surgery (18%). Considering the outcome of patients, defined as favorable or unfavorable (death, change in antibiotic regimen or reoperation with or without modification of antibiotherapy), Meyer et al. 19 reported a large number of events (for example, a need for gastroscopy or CT scan), but these were not necessarily linked to very severe complications in the patients. Indeed, the number of futile events in that study is unclear. This may partly explain why the authors observed a relationship between events and CRP concentrations, but not between complications and CRP concentrations.

Our study also has certain limitations. First, we excluded 25 patients (2%) from the final analysis due to a lack of data; this was necessary because of the retrospective nature of the study. Although a prospective study design would have prevented this issue, the percentage of patients excluded was still low. Similarly, the absence of a fixed rule for ICU admission of these patients may have biased the results. Nevertheless, the relatively long study period of 2 years should have limited the potential effects of periods of lower or higher ICU admission rates. Second, the group of patients with an unfavorable outcome was heterogeneous (with outcomes including death, a change in antibiotic regimen and new surgery), although we analyzed these subgroups separately to determine the delta concentrations (Figure 3). However, larger, separate groups will be needed to confirm our results. Third, the CRP concentrations may have been underestimated because of hemodilution due to fluid infusion during surgery or during the ICU stay and also vascular leakage, as observed in critically ill patients, and especially septic patients. This last point could be assessed based on the ratio of albumin or protein concentration to the CRP concentration 33. Regrettably, though, we do not have data on albumin concentrations for our patients because this parameter is not measured routinely in our ICU patients. Fourth, we measured the concentrations of CRP and certain other inflammatory markers but did not measure other markers, such as CD64 and triggering receptor expressed on myeloid cells (TREM). These other biomarkers may have greater sensitivity for outcome in this particular population 34 but are not routinely measured and need to be further validated. We preferred to study CRP concentrations because they are easy, rapid, and inexpensive to determine and very reproducible.

Fifth, given the design of the study (retrospective and not blind to the CRP values), we cannot exclude the possibility that the judgment of the clinicians was influenced by the CRP values, including their decision to change the antibiotic regimen or to perform new surgery or drainage. Sixth, due to the retrospective design of our study, we used the definition of SIRS, rather than the new definition of sepsis 2 or the “quick” SOFA score (including hypotension, altered mentation, and tachypnea). Other studies with comparisons between CRP concentrations and the “quick” SOFA criteria are needed to prompt clinicians to further evaluate patients for the presence of infection and/or organ dysfunction.

In conclusion, a CRP threshold of 41 mg/L during the first day of ICU admission after major abdominal surgery is a good predictor of an unfavorable outcome. However, no changes in the CRP concentration over time (delta CRP), especially at day 4 or 5, can help to identify patients at risk of death.

## AUTHOR CONTRIBUTIONS

Sapin F and Piagnerelli M were in charge of data collection. Sapin F, Biston P and Piagnerelli M collaborated on the data analysis. All of the authors participated in the study design, study coordination and drafting of the manuscript.

## Figures and Tables

**Table 1:** Demographic, clinical and biological characteristics of all patients admitted to the ICU after abdominal surgery (n=308).

Age (years)	63 [52-72]
Sex (M/F)	50.6 /49.4
Apache II score	11 [6-16]
SOFA score	3 [1-4]
Temperature (°C)	36.0 [35.1-37.1]
Mean arterial blood pressure (mmHg)	69 [61-82]
Height (cm)	168 [160-175]
Weight (kg)	73 [61-95]
NorE dosage (mcg/kg h-) (% of patients)	0.26 [0.15-0.48] (14%)
Diuresis on first day (mL)	1010 [650-1600]
PaO_2_/FiO_2_	290 [205-378]
Hb (g/dL)	10.9 [9.3-12.7]
WBCs (10^3^/mm^3^)	9745 [6010-14600]
Neutrophils (10^3^/mm^3^)	9.14 [5.43-14.26]
Platelets (10^3^/mm^3^)	216 [159-282]
Fibrinogen (g/L)	3.72 [2.62-5.26]
Urea (mg/dL)	35 [25-52]
Creatinine (mg/dL)	0. 99 [0.72-1.30]
Bilirubin (mg/dL)	0.7 [0.5-1.2]
CRP (mg/L)	28 [7-163]
Lactate (mEq/L)	19 [12-31]
ICU length of stay (days)	3 [2-5]
Hospital length of stay (days)	16 [10-32]
Type of surgery, n (%)	Colorectal: 146 (47)
	Bariatric: 42 (14)
	Hepatobiliary-pancreas: 45 (15)
	Esophageal-stomach: 32 (10)
	Urology: 31 (10)
	Gynecology: 12 (4)
ICU death, n (%)	25 (8)

NorE: norepinephrine; Hb: hemoglobin; WBCs: white blood cells (normal range: 4.00-10.00 x 10^3^/mm^3^). Neutrophilia: normal range: 40.0-80.00%. Results shown as median [interquartile range] unless stated otherwise.

**Table 2:** Comparisons of demographic, clinical and biological characteristics between patients with scheduled surgery (n=176) and patients with emergency surgery (n=132).

	Scheduled surgery	Emergency surgery	*p*-value
**Age (years)**	62[52-69]	63[51-78]	0.22
**Sex (M/F)**	93/83	62/70	0.37
**Apache II score**	9[6-13]	14[10-20]	<0.0001
**SOFA score**	2[1-3]	4[1-6]	<0.0001
**Temperature (°C)**	35.8[35.1-35.9]	36.0[35.2-37.4]	0.01
**Mean arterial blood pressure (mmHg)**	73[62-82]	66[60-79]	0.04
**Height (cm)**	166[160-173]	168[160-175]	0.66
**Weight (kg)**	75[67-103]	70[60-85]	0.0003
**NorE dosage (mcg/kg h) (% of patients)**	0.14[0.09-0.20] (6)	0.34[0.18-0.55] (25)	0.01
**Diuresis on first day (mL)**	1155[798-1766]	820[550-1265]	<0.0001
**PaO_2_**/FiO_2_	307[230-403]	267[165-355]	0.002
**Hb (g/dL)**	11.0[9.5-12.6]	10.5[9.0-12.7]	0.20
**WBCs (10^3^**/mm^3^)	11.8[8.1-22.6]	11.7[4.5-17.3]	0.87
**Neutrophils (10^3^**/mm^3^)	9.7[6.4-13.7]	8.9[4.7-14.6]	0.81
**Platelets (10^3^**/mm^3^)	217[160-268]	216[159-310]	0.6
**Fibrinogen (g/L)**	3.2[2.5-4.2]	4.5[3.2-7.5]	<0.0001
**Urea (mg/dL)**	34[26-38]	37[33-42]	<0.0001
**Creatinine (mg/dL)**	0.88[0.70-1.10]	1.08[0.80-1.82]	<0.0001
**Bilirubin (mg/dL)**	0.62[0.44-1.00]	0.80[0.60-1.30]	0.02
**CRP (mg/L)**	8[4-21]	171[64-322]	<0.0001
**Lactate (mEq/L)**	13.5[9.0-21.8]	14.5[8.0-23.4]	0.86
**ICU length of stay (days)**	2[2-4]	4[3-7]	<0.0001
**Hospital length of stay (days)**	14[8-25]	24[13-48]	<0.0001
**ICU death**	7	18	0.001

NorE: norepinephrine; Hb: hemoglobin; WBCs: white blood cells (normal range: 4.00-10.00 x 10^3^/mm^3^). Neutrophilia: normal range: 40.0-80.00%. Results shown as median [interquartile range] unless stated otherwise.

**Table 3:** Comparisons of demographic, clinical and biological characteristics between patients with favorable (n=222) and unfavorable (n=86) ICU outcomes.

	Favorable	Unfavorable	*p*-value
Age (years)	63[56-72]	63[50-72]	0.19
Sex (M/F)	108/114	46/40	0.45
Apache II score	9[6-14]	16[12-22]	<0.001
SOFA score	2[1-4]	4[3-8]	<0.001
Temperature (°C)	35.8[35.1-37.0]	36.4[35.1-37.4]	0.017
Mean arterial blood pressure (mmHg)	73[62-83]	64[59-76]	0.001
Height (cm)	166[160-174]	170[160-175]	0.4
Weight (kg)	73.5[63.5-99.5]	70.0[60.0-87.0]	0.1
NorE dosage (mcg/kg h) (% of patients)	0.2[0.12-0.23] (32.6)	0.39[0.17-0.59] (6.7)	0.024
Diuresis on first day (mL)	1100[710-1670]	850[558-1290]	0.002
PaO_2_/FiO_2_	315[234-426]	220[143-313]	<0.001
Hb (g/dL)	11.0[9.5-12.8]	10.7[8.8-12.1]	0.08
WBCs (10^3^/mm^3^)	11.89[8.60-15.98]	10.32[4.34-17.00]	0.12
Neutrophils (10^3^/mm^3^)	9.53[6.20-14.03]	8.02[2.47-14.66]	0.20
Platelets (10^3^/mm^3^)	216[163-276]	215[142-282]	0.62
Fibrinogen (g/L)	3.65[2.55-5.20]	4.26[2.67-6.16]	0.24
Urea (mg/dL)	32[23-45]	51[31-82]	<0.001
Creatinine (mg/dL)	0.90[0.70-1.12]	1.06[0.80-2.00]	0.002
Bilirubin (mg/dL)	0.70[0.50-1.12]	0.80[0.49-1.39]	0.36
CRP (mg/L)	14[5-86]	141[45-315]	<0.001
Lactate (mEq/L)	15.0[10.0-24.5]	26.0[18.0-42.8]	<0.001
ICU length of stay (days)	2[2-4]	7[4-15]	<0.001
Hospital length of stay (days)	15[9-25]	35[14-53]	<0.001
ICU death	0	25	<0.001

NorE: norepinephrine; Hb: hemoglobin; WBCs: white blood cells (normal range: 4.00-10.00 x 10^3^/mm^3^). Neutrophilia: normal range: 40.0-80.00%. Results shown as median [interquartile range] unless stated otherwise.
